# A Serological Survey of Measles and Rubella Antibodies among Different Age Groups in Eastern China

**DOI:** 10.3390/vaccines12080842

**Published:** 2024-07-25

**Authors:** Rui Yan, Hanqing He, Xuan Deng, Yang Zhou, Xuewen Tang, Yao Zhu, Hui Liang, Yaping Chen, Mengya Yang, Yuxia Du, Can Chen, Jiaxin Chen, Shigui Yang, RIDPHE Group

**Affiliations:** 1 Department of Emergency Medicine, Department of Epidemiology and Biostatistics, School of Public Health, Zhejiang University School of Medicine, Second Affiliated Hospital, Hangzhou 310000, China; ryan@cdc.zj.cn (R.Y.); chencan@zju.edu.cn (C.C.); 13606568178@163.com (J.C.); 2 Zhejiang Provincial Center for Disease Control and Prevention, Hangzhou 310057, Chinahliang@cdc.zj.cn (H.L.); 22118924@zju.edu.cn (M.Y.)

**Keywords:** measles virus, rubella virus, antibody level, herd immunity

## Abstract

Background: Measles and rubella are vaccine-preventable diseases targeted for elimination in most World Health Organization regions, and China is considered to have momentum towards measles elimination. Therefore, this study aimed to assess the population immunity levels against measles and rubella in Zhejiang Province in China in order to provide valuable insights for informing future public health measures and contributing to the ongoing global campaign against these diseases. Materials and methods: A cross-sectional serological survey was conducted in 2022. A total of 2740 blood samples were collected from healthy individuals spanning the age range of 0–59 years, representing diverse demographic strata across 11 prefectures in Zhejiang Province in China. The sera were tested for measles and rubella IgG antibodies to determine positivity rates and geometric mean concentrations (GMCs). Results: The overall positivity rate for the measles IgG antibody was 85.3%, with a GMC of 588.30 mIU/mL. The positivity rate for the rubella IgG antibody was 70.9%, and the GMC was 35.30 IU/mL. Measles IgG antibody positivity rates across the 0–11 months, 12–23 months, 24–35 months, 3–5 years, 6–9 years, 10–14 years, 15–19 years, 20–29 years, and 30–59 years age groups were 63.1%, 92.5%, 97.0%, 94.0%, 85.8%, 77.3%, 86.9%, 84.9%, and 88.7%, respectively (trend *χ*^2^ = 118.34, *p* < 0.001). Correspondingly, rubella antibody positivity rates for these same age brackets were 55.9%, 87.9%, 94.7%, 88.2%, 69.9%, 54.2%, 72.6%, 67.5%, and 74.3% (trend *χ*^2^ = 199.18, *p* < 0.001). Both univariate and multivariate analyses consistently demonstrated that age, immunization history, and differing economic levels were significant factors contributing to variations in antibody levels. Conclusions: The seroprevalence of measles and rubella was lower than that required for herd immunity. Periodic vaccination campaigns should be launched to increase immunity.

## 1. Introduction

Both measles and rubella are acute virus-induced illnesses and are characterized by fever and rash [1,2]. Vaccination has drastically reduced global measles-related deaths, with an 83% drop between 2000 and 2021 worldwide [3]. From 2021 to 2022, the estimated number of measles cases increased by 18%, and the number of countries experiencing large or disruptive outbreaks increased from 22 to 37 [4]. The number of reported rubella cases declined by 97%, from 670,894 cases in 102 countries in 2000 to 14,621 cases in 151 countries in 2018 [5]. As of January 2024, 175 of 194 countries had introduced rubella vaccines, and the global coverage was estimated at 69% [6].

In Zhejiang Province, which is located along the eastern coast of China and comprises 11 prefecture-level divisions, the measles vaccine was introduced in 1967, and routine immunization has been in place since 1978. A mandatory two-dose vaccination schedule was implemented in 1986, with doses administered at the ages of eight months and seven years [7]. The rubella-containing vaccine (RCV) was first licensed in Zhejiang in 1993 [8]. The coverage rate for the first dose of the measles-containing vaccine (MCV) among children reaching 12 months of age has been at least 95% in Zhejiang since 1999 [5]. Furthermore, since 2006, more than 95% of local children receive two doses of MCV by the time they reach 24 months of age [9]. In 2008, China extended the Expanded Program on Immunization (EPI) to include RCV, which began to be offered without charge in routine immunization services. The measles and rubella vaccine (MR) was administered as a first dose at 8 months, and the measles–mumps–rubella vaccine (MMR) was administered as a second dose at 18–24 months. The Zhejiang Provincial Government set up an additional vaccination campaign for MR covering ninth-grade students, irrespective of their immunization status, from 2011 [5]. Since then, approximately 450,000 students have been vaccinated annually, with the MR coverage rate exceeding 95% [10]. In 2019, the Zhejiang Provincial Government changed the MCV from MR to MMR; currently, there is a three-dose policy (8 months, 18–24 months, and 13–14 years) for MMR in Zhejiang Province [11]. About 50 years ago, measles was a very common infectious disease across Zhejiang Province, similar to many other regions globally. Indeed, almost every child under the age of five was infected with it [9]. Since measles was included in the National Notifiable Diseases Reporting System (NNDRS) in 1951, the incidence rate has dropped by more than 99% from the highest value of 3050.69 per 100,000 in 1959 to 0.02 per 100,000 in 2023. Rubella has been a notable disease in Zhejiang Province since 1993. Initially, it was monitored via sentinel surveillance in 16 prefectures. However, following its incorporation into the NNDRS in 2004, there has been a substantial decrease in the incidence rate, which plummeted from a high of 37.87 per 100,000 in 2008 to 0.05 per 100,000 by 2023.

Between 2020 and 2022, the measures implemented by the Chinese government to control the COVID-19 pandemic, including temporary lockdowns, mask wearing, social distancing, COVID-19 vaccination, improved personal hygiene, and restricted travel, may have proven effective in preventing the spread of infectious respiratory diseases such as measles [12]. When carrying out large-scale COVID-19 vaccination campaigns and temporary lockdowns, there might have been some impact on routine immunization efforts to a certain extent. However, in accordance with Zhejiang Province’s vaccination plan guidelines, when an individual is required to receive more than two vaccines simultaneously, priority should be given to administering MCV [11]. From 2020 to 2022, two rounds of supplementary immunization activities (SIAs) of the measles-containing vaccine (MCV) were still conducted annually. In the post-COVID-19 era, to sustain the low incidence rate of measles, it is imperative to consistently maintain rigorous reinforcement of all routine measles control measures without any letup.

Both measles and rubella have been specifically recognized and targeted for elimination by the World Health Organization (WHO). As of May 2024, a total of 78 countries worldwide have verified the elimination of measles, while 94 countries have verified the elimination of rubella [13]. Mathematical models have estimated that the herd immunity thresholds necessary to interrupt the transmission of measles and rubella are 93–95% and 83–85%, respectively [14]. In alignment with Strategic Objective 1 (SO1) of the regional strategic plan for measles and rubella elimination, the goal is to achieve and maintain high population immunity, ensuring at least 95% vaccination coverage with two doses of measles- and rubella-containing vaccines in every district of every country [15]. In this study, we monitored measles and rubella antibodies among healthy individuals living in Zhejiang Province in order to investigate whether the WHO-specified herd immunity levels had been achieved.

## 2. Materials and Methods

### 2.1. Study Participants

A population-based cross-sectional surveillance study was conducted in Zhejiang Province between May and October 2022. The study participants were local residents aged 0–59 years who had lived in Zhejiang Province for more than 6 months. Participants were excluded if they had an acute disease or immunodeficiency, a history of immune disease, or a history of immunosuppressive agent use. Study participants were also ineligible if they had received blood products or immunoglobulins during the previous 3 months. The vaccination status of participants under the age of 15 years was established through a review of immunization record books or the Immunization Information System of Zhejiang Province. For those aged 14 years and older, the determination of vaccination status largely relied on the participants’ personal recollections. The disease status of all the participants was derived mainly from the recall of the participants themselves.

According to the formula for calculating the sample size of a cross-sectional survey, the antibody positivity rates for measles and rubella were set at 85% [5,16], the design effect was set at 2, and the loss to follow-up rate was set at 10%. The survey was conducted on a total of 2578 participants. These participants were selected across one county (city or district) within each of the 11 prefecture-level cities in Zhejiang Province, with a minimum of 240 participants sourced from each chosen county (city or district). Adherence to the research protocol was paramount, as was obtaining informed consent. We meticulously implemented established inclusion and exclusion criteria for participants, ensuring not only a balanced gender representation but also that the age distribution fulfilled the requirement of a minimum of nine respondents per age bracket: 0–11 months, 12–23 months, 24–35 months, 3–5 years, 6–9 years, 10–14 years, 15–19 years, 20–29 years, and 30–59 years. The investigation was primarily undertaken at the county-level Centers for Disease Control and Prevention and Community Health Service Centers, frequently aligning with routine community health check-ups. When initial sampling failed to provide adequate numbers for particular age categories, supplementary surveys were administered to expand the reach within the wider community.

### 2.2. Laboratory Tests

A 3–5 mL blood sample was obtained via the median cubital vein, followed by centrifugation after a minimum interval of 1 h, with the serum then transferred to plain polypropylene tubes and stored at −20 °C. Serological tests were performed at the Laboratory of Product Development Department, Dalian Sinovac Company. This laboratory met the accreditation criteria of the WHO National Measles Laboratories. Serum IgG antibodies against measles and rubella were measured by enzyme-linked immunosorbent assays (ELISAs) using commercially available kits (Virion/Serion ESR 102G/ESR129G, Wurzburg, Germany). The cutoff values and final results were based on the qualitative criteria outlined by the manufacturer, and standard duplicate controls and negative controls were used for every plate. A measles IgG antibody concentration of ≥200 mIU/mL was considered positive, a concentration of <150 was considered negative, and a concentration between 150 and 200 was considered equivocal. A rubella IgG antibody concentration of ≥20 IU/mL was considered positive, a concentration of <10 was considered negative, and a concentration between 10 and 20 was considered equivocal.

### 2.3. Statistical Analysis

To obtain unbiased geometric mean concentrations (GMCs) for measles and rubella, values below the measles detection threshold (50 mIU/mL) were assigned half the threshold value (25 mIU/mL). Similarly, values below the rubella detection threshold (2 IU/mL) were assigned half the threshold value (1 IU/mL). The 95% confidence interval (CI) for the seroprevalence of measles and rubella was estimated using the Clopper–Pearson exact binomial interval. The geometric mean concentrations (GMCs) of measles and rubella were summarized by the medians and interquartile ranges [M (Q1–Q3)]. For comparisons of seroprevalence between groups with ordered categories, the trend chi-square test was employed, whereas for seroprevalence comparisons among other groups, the standard chi-square test was utilized. The GMC comparison between two categorical variables, such as gender and residence, was conducted using the Wilcoxon rank-sum test, whereas GMC comparisons involving other multicategorical variables were analyzed using the Kruskal–Wallis H test.

Univariate and multivariate analyses were carried out using logistic regression models, and ORs and 95% CIs were used to describe the influence of each factor on the seroprevalence of measles and rubella. In the multivariate regression analysis, the ‘backward elimination’ method was used to construct the model, and the Hosmer–Lemeshow goodness-of-fit test was applied to evaluate the adequacy of the model fit. All statistical tests were two-sided and were considered statistically significant at *p* < 0.05. Data entry and checks were conducted using EpiData (version 3.1), and data analysis was performed using Microsoft Office Excel 2017 and SPSS for Windows (version 18.0).

In this study, women aged 15–49 years were considered as women of reproductive age [17]. Based on the level of economic development, the 11 prefecture-level cities in Zhejiang Province were classified into three categories: developed (Hangzhou, Ningbo, and Wenzhou), moderately developed (Shaoxing, Jiaxing, Taizhou, and Jinhua), and general regions (Huzhou, Zhoushan, Quzhou, and Lishui).

### 2.4. Ethical Statement

This study was approved by the Ethics Review Committee of the Zhejiang Provincial Center for Disease Control and Prevention (approval number: 2022-012-01; approval date: 30 March 2022). All participants signed a written consent form, and serum samples were anonymized and encrypted during the study.

## 3. Results

### 3.1. Basic Characteristics of the Study Group

We recruited a total of 2758 participants, of whom 2740 agreed to participate in our investigation or provided suitable blood samples, and these participants underwent testing for specific anti-measles IgG and specific anti-rubella IgG antibodies. There were 1227 males and 1513 females, with a sex ratio of 1:1.23. The median age (Q1–Q3) was 11 (5–18) years. Notably, there were 589 women of reproductive age (15–49 years), accounting for 21.5% of the cohort. A total of 1576 individuals (57.5%) were registered as urban residents. Furthermore, 27 individuals (1.0%) had a medical history of measles, and an even smaller fraction of 0.4% (12 individuals) had a history of rubella. When focusing on the segment of children and adolescents aged between 0 and 14 years, 93.4% (1646 of 1762) had a history of vaccination with MCV, while 93.4% (1643 of 1762) had a history of vaccination with RCV.

### 3.2. Seroprevalence and GMCs of Measles and Rubella Antibodies

#### 3.2.1. Overview

Out of the 2740 participants, 2337 (85.3%) were seropositive and 403 (14.7%) were seronegative for the measles virus, whereas 1904 (70.9%) were seropositive and 798 (29.1%) were seronegative for the rubella virus. The median (Q1–Q3) GMC for measles was 588.30 (302.33, 1155.25) mIU/mL, while for rubella the median (Q1–Q3) GMC was 35.30 (17.35, 65.70) IU/mL. The median (Q1–Q3) age was 11 years (5–18 years), ranging from 0 to 59 years, at the time of serum collection.

#### 3.2.2. Age Distribution

**Figure 1:**
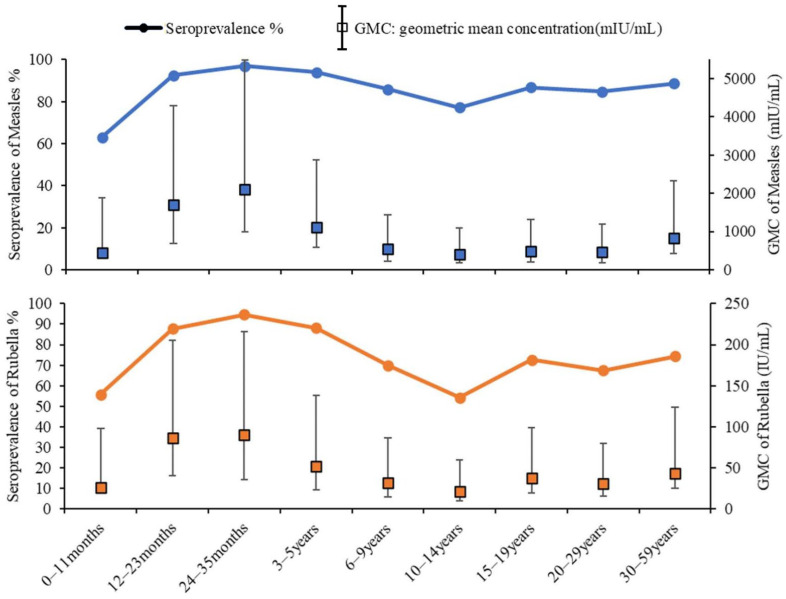
Seroprevalence and geometric mean concentrations of measles and rubella antibodies among different age groups in Zhejiang Province, 2022. GMC: geometric mean concentration.

The antibody positivity rates of measles for the 0–11 months, 12–23 months, 24–35 months, 3–5 years, 6–9 years, 10–14 years, 15–19 years, 20–29 years, and 30–59 years age groups were, respectively, 63.1%, 92.5%, 97.0%, 94.0%, 85.8%, 77.3%, 86.9%, 84.9% and 88.7% (trend *χ*^2^ = 118.34, *p* < 0.001), whereas the GMCs of measles were, respectively, 8.26, 78.27, 61.20, 30.66, 19.03, 35.13, 33.24, 45.93, and 31.94 mIU/mL (*H* = 746.43, *p* < 0.001). The rubella antibody positivity rates in the aforementioned age groups were as follows: 55.9%, 87.9%, 94.7%, 88.2%, 69.9%, 54.2%, 72.6%, 67.5%, and 74.3% (trend *χ*^2^ = 199.18, *p* < 0.001); correspondingly, the GMCs for rubella were 26.30, 86.90, 90.00, 52.45, 32.30, 21.80, 38.10, 31.30, and 43.80 IU/mL (*H* = 400.84, *p* < 0.001) (Figure 1).

#### 3.2.3. Gender Distribution, Urban versus Rural Residence, and Immunization History Distribution

The IgG seropositivity rates for measles were 83.7% in males and 86.6% in females (*χ*^2^ = 4.49, *p* = 0.034), with GMCs of 548.10 mIU/mL and 620.30 mIU/mL, respectively (*Z* = −2.10, *p* = 0.036). For rubella, the IgG seropositivity rates were 70.82% in males and 70.92% in females (*χ*^2^ < 0.01, *p* = 0.956), with GMCs of 35.00 IU/mL and 36.20 IU/mL, respectively (*Z* = −0.21, *p* = 0.838). The rubella IgG seropositivity rates were 68.8% among women of childbearing age and 78.3% in their age-matched male counterparts (*χ*^2^ = 8.54, *p* = 0.003), with GMCs of 35.60 IU/mL and 40.50 IU/mL, respectively (*Z* = −2.23, *p* = 0.026).

The measles IgG seropositivity rates were 85.2% among individuals with urban residency and 85.4% among those with rural residency (*χ*^2^ = 0.02, *p* = 0.896), with GMCs of 595.75 mIU/mL and 580.65 mIU/mL, respectively (*Z* = −0.57, *p* = 0.570). For rubella IgG, the seropositivity rates were 69.9% and 72.3% in the urban and rural populations, respectively (*χ*^2^ = 1.86, *p* = 0.173), with corresponding GMCs of 34.80 IU/mL and 36.75 IU/mL, respectively (*Z* = −1.04, *p* = 0.297).

The measles IgG seropositivity rates among individuals with MCV vaccination histories of zero doses, one dose, two doses, three doses, and unknown were 80.0%, 88.9%, 85.8%, 88.2%, and 85.6%, respectively (*χ*^2^ = 12.82, *p* = 0.012), with geometric mean concentrations (GMCs) of 574.80, 1005.20, 593.50, 523.55, and 564.80 IU/mL (*H* = 27.48, *p* < 0.001). For rubella IgG, the seropositivity rates among those with RCV vaccination histories of zero doses, one dose, two doses, three doses, and unknown were 67.7%, 76.4%, 70.9%, 75.3%, and 69.1%, respectively (*χ*^2^ = 8.12, *p* = 0.087), with corresponding GMCs of 34.90, 43.10, 34.90, 39.20, and 33.25 IU/mL (*H* = 12.08, *p* = 0.017).

The IgG antibody positivity rates for measles and rubella among the survey participants in 11 prefecture-level cities ranged from 69.17% to 94.69% (*χ*^2^ = 100.38, *p* < 0.001) and from 46.67% to 83.27% (*χ*^2^ = 114.03, *p* < 0.001), respectively.

### 3.3. Univariate and Multivariate Analysis of the Factors Influencing Measles Antibody Positivity Rates

Univariate analysis revealed statistically significant variations in measles antibody positivity rates across genders, age groups, regions, and histories of MCV immunization (Table 1 and Table 2). Moreover, this analysis specifically indicated significant differences in the GMCs of measles antibodies among individuals characterized by sex, age group, region, and MCV immunization (Table 1).

The results of the Hosmer–Lemeshow goodness-of-fit test for the multivariate regression model using the ‘backward elimination’ method showed a *p*-value of 0.159, suggesting a satisfactory fit of the model. The outcomes of the multivariate regression models identified gender, age groups, regions, and MCV vaccination status as factors influencing the seroprevalence of measles (Table 2).

### 3.4. Univariate and Multivariate Analysis of the Factors Influencing Rubella Antibody Positivity Rates

Univariate analysis showed that the positivity rates of rubella IgG antibodies were statistically different among the different age groups and regions. Moreover, there were statistically significant variations in the GMCs of rubella IgG antibodies across different age groups, regions, history of rubella infection, and RCV inoculation doses (Table 3 and Table 4).

In the multivariate regression model assessing rubella antibody seropositivity, the Hosmer–Lemeshow goodness-of-fit test resulted in a *p*-value of 0.521 for the model using the ‘backward elimination’ method. The *p*-value, being greater than 0.05, suggested that the model adequately fit the data according to the Hosmer–Lemeshow criterion. The results of the multivariate analysis showed that there were statistically significant differences in the positivity rates of rubella IgG antibodies among people of different regions and age groups and with different immunization histories (Table 4).

## 4. Discussion

This study aimed to estimate the seroprevalence of measles and rubella in the general population of Zhejiang Province in China. We found that approximately 85% of our study population had serological proof of immunity against the measles virus, whereas 70% had serological proof of immunity against the rubella virus, indicating that approximately 15% were susceptible to measles and 30% were susceptible to rubella. Similar seropositivity levels have been observed in other provinces of China and in European serosurveys. In 2022, among children aged 0–6 years in Sichuan Province, 81.35% tested positive for measles antibodies and 69.68% tested positive for rubella antibodies [18]. Comparatively, in a 2020 survey of the healthy population in Guangzhou City, 89.99% of the participants were measles antibody-positive and 80.16% were rubella antibody-positive [19]. European serosurveys showed that 89.3% of the general population in Greece were measles antibody-positive, while in Germany the measles seroprevalence in healthy participants was 89.9% and the rubella seroprevalence was 90%. In Spain, the seroprevalence of measles and rubella was 92.1% and 94.4% [20,21,22]. Considering that the WHO endorses the widely accepted target of achieving at least 95% vaccination coverage with two doses of measles-containing vaccines and 85% coverage with two doses of rubella-containing vaccines to effectively control the spread of these viruses, our findings reveal an immunity gap against measles and rubella in the Zhejiang population [23]. Given the insufficiently robust population immunity barrier against measles and rubella, vigilance must be maintained against the potential transmission of these viruses, even during periods of low numbers of reported cases.

In this survey, the patterns of measles and rubella antibody levels across different age groups, represented by seropositivity rates and GMCs, consistently demonstrated similar trends. These levels were found to be lowest among infants aged 0–11 months, peaking in children aged 24–35 months, followed by a decline until a subsequent rise in adolescents aged 10–14 years. Infants aged 0–11 months exhibited the lowest levels of measles and rubella antibodies, primarily due to the decline in maternally derived antibodies against these viruses to their minimum values by 6 months of age [1,2]. In the present scenario in Zhejiang Province, the first dose of the MMR vaccine is scheduled for administration at 8 months of age. However, many children fail to receive timely inoculation due to illnesses or for other reasons [24]. Children aged 24–35 months displayed the highest levels of measles and rubella antibodies because, within this age group, they had recently completed two doses of the MMR vaccine in accordance with the recommended immunization schedule. Following two doses of measles- and rubella-containing vaccines, there was a decline in both the seropositivity rates and GMCs of measles and rubella antibodies observed from age 3 to 14 years. In Zhejiang Province, following the implementation of the third dose of MMR vaccination among ninth-grade students, there was a significant increase in IgG antibody positivity rates for measles and rubella among individuals aged 15–19, indicating that an additional dose of MMR is necessary for this population in Zhejiang [25]. The WHO’s measles and rubella elimination strategy stipulates that a supplementary dose of a vaccine containing both measles and rubella components for adolescents and adults is crucial for accelerating the elimination of rubella and congenital rubella syndrome (CRS) [26]. This study further revealed that the rubella IgG antibody positivity rate among women of childbearing age was only 68.76%, falling short of the threshold for rubella herd immunity [14]. RV infection during early pregnancy can lead to CRS in newborns. Therefore, it is recommended that women with negative rubella antibodies and plans for conception receive a rubella-containing vaccine to mitigate the risks of rubella infection and CRS occurrence in newborns.

This study showed that individuals with a history of either MCV or RCV vaccination exhibited significantly higher measles/rubella antibody positivity rates and GMCs than those with no history of immunization, indicating that vaccination is highly effective in enhancing and solidifying population immunity barriers [27]. However, the seropositivity rates and GMCs of measles and rubella did not increase with the number of doses administered, which might be attributed to the secondary dose of MMR inducing lower levels of antibodies compared to the initial MMR vaccination [28]. Among the study subjects who were unvaccinated, 80% and 67% were still seropositive for measles and rubella antibodies, respectively. A portion of them might have previously contracted measles or rubella, or bias could have been introduced due to recall, but the high positivity rates of measles and rubella antibodies also indicated a robust immune barrier among Zhejiang Province residents.

In addition, cities with higher levels of economic development demonstrated lower measles and rubella antibody levels and GMCs than those with slightly higher or moderate levels of economic development. This may be because in economically prosperous cities people tend to pay greater attention to adverse events following vaccination, leading to increased vaccine hesitancy [29,30]. Despite the decline in measles and rubella incidence in Zhejiang Province in recent years and no reported outbreaks of either disease [25], the increasing number of individuals refusing or delaying vaccinations has contributed to a decrease in antibody levels for both measles and rubella.

Our study has some limitations. Firstly, samples obtained conveniently during health check-ups may differ from those obtained from the broader community population, potentially impacting the representativeness of the sample. Secondly, the reliance on self-reported disease and vaccination histories from adults introduces the possibility of recall bias, which could have affected our study outcomes. Thirdly, the demographic data collected were limited in scope, omitting consideration of other potential factors influencing measles and rubella IgG antibody seropositivity. Additionally, there was no possibility of distinguishing between vaccination-induced immunity and immunity induced by natural infection during laboratory analysis. Furthermore, while the neutralization test (NT) is esteemed as the gold standard for measles and rubella immunity assessment, ELISAs are more pragmatic and commonly used, especially in extensive serological surveys. A significant correlation exists between NT and ELISA outcomes for seropositive cases, validating the appropriateness of IgG ELISAs for measles and rubella immunity evaluations [31]. Despite these methodological considerations, our study still furnishes a valuable approximation of the measles and rubella immunity deficiency in Zhejiang Province.

## 5. Conclusions

Measles and rubella are vaccine-preventable diseases that are targeted for elimination in most WHO regions. China is considered to have momentum towards measles elimination [32]. Unfortunately, the seroprevalences of measles and rubella found in this study were lower than those required for herd immunity. Measles and rubella seroprevalence decreased over time after vaccination, and administration of the MMR vaccine to junior high-school students effectively boosted these antibody levels. To increase immunity, vaccination campaigns must be periodically launched to raise awareness of the benefits of vaccines and address the issue of vaccine hesitancy.

## Figures and Tables

**Table 1:** Univariate analysis of the factors influencing measles seroprevalence and GMCs.

Characteristics	Total	Seroprevalence				GMCs				
		%	95% CI	*χ* ^2^	Sig.	Median	P_25_	P_75_	Z/H	Sig.
Gender				4.49	0.034				−2.10 ^b^	0.036
Male	1227	83.7	81.53–85.66			548.10	282.50	1145.40		
Female	1513	86.6	84.77–88.21			620.30	318.05	1165.55		
Age groups				118.34 ^a^	<0.001				505.95 ^c^	<0.001
0–11 months	111	63.1	53.79–71.46			445.30	75.10	1428.90		
12–23 months	107	92.5	85.93–96.16			1696.10	1008.20	2596.40		
24–35 months	133	97.0	92.52–98.82			2102.00	1115.60	3385.40		
3–5 years	348	94.0	90.96–96.02			1116.90	523.20	1755.45		
6–9 years	465	85.8	82.34–88.69			541.00	317.80	903.80		
10–14 years	598	77.3	73.73–80.44			397.05	216.00	705.00		
15–19 years	412	86.9	83.29–89.81			493.60	289.05	818.95		
20–29 years	231	84.9	79.66–88.90			458.60	273.90	733.50		
30–59 years	335	88.7	84.82–91.63			829.30	400.50	1495.80		
Regions				33.41 ^a^	<0.001				36.30 ^c^	<0.001
Developed	798	79.3	76.37–81.99			495.15	240	1002.7		
Moderately developed	960	86.8	84.48–88.77			635.35	329.15	1276		
General	982	88.7	86.57–90.53			615.45	332.5	1218.2		
Residence				0.02	0.896				−0.57 ^b^	0.570
Urban	1576	85.2	83.38–86.89			595.75	303.35	1162.70		
Rural	1164	85.4	83.26–87.31			580.65	299.95	1147.63		
History of measles				2.33	0.311				2.27 ^c^	0.321
Yes	27	92.6	76.63–97.94			758.10	409.60	2386.90		
No	2560	85.0	83.61–86.37			588.30	299.93	1154.38		
Unknown	153	88.2	82.18–92.43			581.70	343.45	1120.90		
Vaccination with MCV				12.82	0.012				27.48 ^c^	<0.001
0 doses	409	80.0	75.8–83.54			547.80	257.65	1062.35		
1 dose	162	88.9	83.12–92.86			1005.20	385.43	1833.10		
2 doses	1575	85.8	84.03–87.47			593.50	315.10	1219.30		
3 doses	212	88.2	83.17–91.89			523.55	294.58	867.73		
Unknown	382	85.6	81.72–88.77			564.80	300.15	1048.18		

Notes: ^a^ Linear-by-linear association in the chi-square test; ^b^ Wilcoxon rank-sum test; ^c^ Kruskal–Wallis H test.

**Table 2:** Univariate and multivariate analysis of variables and their association with measles seroprevalence.

Variables	Univariate Analysis		Multivariate Analysis	
	OR	95% CI	OR	95% CI
Gender (Male)	1.26	1.01–1.55	1.28	1.02–1.60
Age groups (0–11 months)				
12–23 months	7.25	3.20–16.40	5.49	2.31–13.01
24–35 months	18.89	6.49–54.91	15.74	5.06–48.97
3–5 years	9.12	5.07–16.38	8.05	3.88–16.65
6–9 years	3.54	2.22–5.63	2.97	1.57–5.59
10–14 years	1.99	1.29–3.05	1.63	0.90–2.94
15–19 years	3.88	2.40–6.27	3.37	1.91–5.94
20–29 years	3.28	1.93–5.55	4.08	2.21–7.50
30–59 years	4.58	2.74–7.64	6.02	3.33–10.85
Regions (Developed)				
Moderately developed	1.71	1.32–2.20	1.73	1.33–2.24
General	2.05	1.57–2.65	2.16	1.64–2.83
Residence (Urban)	1.01	0.81–1.25		
History of measles (Yes)				
No	0.45	0.10–1.92		
Unknown	0.60	0.13–2.74		
Vaccination with MCV (0 doses)				
1 dose	2.01	1.16–3.46	2.87	1.54–5.33
2 doses	1.52	1.14–2.01	1.69	1.06–2.68
3 doses	1.88	1.15–3.03	2.51	1.41–4.47
Unknown	1.49	1.02–2.16	1.00	0.65–1.52

**Table 3:** Univariate analysis of the factors influencing rubella seroprevalence and GMCs.

Characteristics	Total	Seroprevalence				GMCs				
		%	95% CI	χ^2^	Sig.	Median	P_25_	P_75_	Z/H	Sig.
Gender				<0.01	0.956				−0.21 ^b^	0.838
Male	1227	70.8	68.21–73.30			35.00	17.10	67.40		
Female	1513	70.9	68.58–73.15			36.20	17.90	66.30		
Age groups				199.18 ^a^	<0.001				400.84 ^c^	<0.001
0–11 months	111	55.9	46.58–64.75			26.30	5.50	71.90		
12–23 months	107	87.9	80.32–92.76			86.90	46.20	118.50		
24–35 months	133	94.7	89.54–97.43			90.00	54.50	126.00		
3–5 years	348	88.2	84.41–91.20			52.45	29.65	85.40		
6–9 years	465	69.9	65.57–73.88			32.30	17.50	53.90		
10–14 years	598	54.2	50.17–58.13			21.80	11.50	37.70		
15–19 years	412	72.6	68.07–76.65			38.10	18.70	61.15		
20–29 years	231	67.5	61.25–73.24			31.30	15.50	48.80		
30–59 years	335	74.3	69.40–78.71			43.80	18.90	80.00		
Regions				42.71 ^a^	<0.001				34.83 ^c^	<0.001
Developed	798	62.0	58.61–65.33			29.00	13.50	59.80		
Moderately developed	960	74.7	71.84–77.34			37.60	19.90	71.30		
General	982	74.3	71.52–76.97			37.85	19.20	68.70		
Residence				1.85	0.173				−1.04 ^b^	0.297
Urban	1576	69.9	67.55–72.07			34.80	17.05	66.45		
Rural	1164	72.3	69.61–74.75			36.75	18.10	66.85		
History of rubella				5.24	0.073				6.36 ^c^	0.042
Yes	12	100.0	75.75–100.00			59.45	34.05	106.90		
No	2574	70.9	69.07–72.58			35.80	17.40	67.10		
Unknown	154	68.8	61.13–75.61			32.35	17.10	55.20		
Vaccination of RCV				8.12	0.087				12.08 ^c^	0.017
0 doses	455	67.7	63.26–71.82			34.90	14.50	63.70		
1 dose	301	76.4	71.30–80.85			43.10	20.60	77.80		
2 doses	1602	71.2	68.89–73.33			34.90	17.40	64.80		
3 doses	89	75.3	65.40–83.07			39.20	20.00	65.70		
Unknown	382	69.1	64.31–73.53			33.25	17.60	67.70		

Notes: ^a^ Linear-by-linear association in the chi-square test; ^b^ Wilcoxon rank-sum test; ^c^ Kruskal–Wallis H test.

**Table 4:** Univariate and multivariate analysis of variables and their association with rubella seroprevalence.

Variables	Univariate Analysis		Multivariate Analysis	
	OR	95% CI	OR	95% CI
Gender (Male)	1.00	0.85–1.18		
Age groups (0–11 months)				
12–23 months	5.71	2.86–11.39	5.20	2.52–10.72
24–35 months	14.23	6.09–33.22	14.78	6.07–35.98
3–5 years	5.92	3.60–9.72	6.59	3.70–11.73
6–9 years	1.83	1.20–2.80	1.97	1.18–3.29
10–14 years	0.93	0.62–1.40	0.90	0.55–1.44
15–19 years	2.09	1.35–3.22	2.16	1.35–3.42
20–29 years	1.64	1.03–2.61	2.12	1.26–3.53
30–59 years	2.29	1.46–3.58	3.01	1.83–4.938
Regions (Developed)				
Moderately developed	1.81	1.47–2.21	1.90	1.53–2.35
General	1.77	1.44–2.17	1.88	1.51–2.32
Residence (Urban)	1.03	0.85–1.23		
History of rubella (No)				
Yes	6.64 × 10^7^	0–		
Unknown	0.91	0.63–1.29		
Vaccination of RCV (0 doses)				
1 dose	1.55	1.11–2.15	1.99	1.34–2.94
2 doses	1.16	0.92–1.45	1.15	0.80–1.64
3 doses	1.45	0.86–2.44	2.04	1.14–3.63
Unknown	1.07	0.79–1.43	0.89	0.64–1.22

## Data Availability

The data presented in this study are available upon request from the corresponding author.
